# Metformin efficacy and tolerance according to genetic polymorphisms of organic cation transporter 1 in Tunisian patients with type 2 diabetes

**DOI:** 10.3389/fendo.2025.1536402

**Published:** 2025-06-30

**Authors:** Fatma Chaker, Ameni Kallel, Nadia Khessairi, Meriem Yazidi, Ibtissem Oueslati, Hiba Allah Chatti, Moncef Feki, Melika Chihaoui

**Affiliations:** ^1^ Department of Endocrinology, Rabta University Hospital, Faculty of Medicine of Tunis, University of Tunis El Manar, Tunis, Tunisia; ^2^ Department of Biochemistry Rabta University Hospital, Faculty of Medicine of Tunis, University of Tunis El Manar, Tunis, Tunisia

**Keywords:** adverse effects, glycemic control, metformin, organic cation transporter 1, pharmacogenetics, SLC22A1, type 2 diabetes

## Abstract

**Purpose:**

Metformin efficacy and tolerance vary at equivalent dose in type 2 diabetes. Inter-individual variability in the response to metformin with approximately 35% of patients failing to achieve initial glycemic control may be explained by genetic polymorphisms that affect the drug’s pharmacokinetics and pharmacodynamics. Differences in the frequencies of pharmacogenomic risk alleles associated with metformin response may also account for interethnic variability in drug effects. The aim of this study was to assess the impact of M420del, R61c, and G401S polymorphisms in the SLC22A1 gene which encodes the organic cation transporter (OCT1) on metformin response and tolerance in a cohort of Tunisian patients with type 2 diabetes.

**Methods:**

This prospective study included 73 newly diagnosed type 2 diabetic patients. Clinical and biological assessments were conducted before and three months after initiation of metformin therapy. Patients were genotyped for the M420del, R61c, and G401S polymorphism of SLC22A1 using Polymerase Chain Reaction (PCR) followed by Restriction Fragment Length Polymorphism (RFLP) analysis. Metformin efficacy was defined as an HbA1c reduction of ≥ 1% and metformin- induced gastrointestinal adverse effects were recorded using a questionnaire.

**Results:**

Thirty-nine patients (53%) were classified as responders to metformin. The M420del, R61C and G401S variants were not significantly associated with metformin efficacy (p: 0.8, p: 0.77, and p: 0.49 respectively). Twenty-seven patients (37%) experienced gastrointestinal adverse effects following metformin initiation. The G401S polymorphism and the haplotype (NoDel) CA were significantly associated with gastrointestinal adverse effects.

**Conclusion:**

In Tunisian type 2 diabetes, the M420del and R61C do not appear to be associated with metformin efficacy or the gastrointestinal intolerance. However, the G401S polymorphism may be implicated in the occurrence of metformine-induced gastrointestinal adverse effects.

## Introduction

Diabetes mellitus is considered as a major public health problem. According to the International Diabetes Federation (IDF Diabetes Atlas), 10.5% of adults were estimated to have diabetes worldwide in 2021 (1). Type 2 diabetes (T2D) is the most common form and accounts for nearly 90% of all diabetes types. In Tunisia, according to the ATERA-Survey study including 10,576 participants (44% men and 56% women) aged 25 to 75 years, the overall prevalence of T2D was 23.0% (2). Metformin offers the best cost-effectiveness ratio with no risk of hypoglycemia and weight gain (3). Some gastrointestinal adverse reactions occur in about 20-30% of patients and are dose-dependent (4). Metformin is a part of the therapeutic panel, ever in the late stage of T2D, in association with other hypoglycemic agents. The efficacy of metformin varies substantially. At the early stage of the disease, approximatively one third of metformin treated patients do not reach their glycemic goals (5). About fifty percent of the oral metformin dose is absorbed passing to blood followed by distribution to different tissues (6). The molecular mechanisms of metformin action are not well known. Investigations in mice demonstrated that metformin inhibits hepatic gluconeogenesis independently of liver kinase B1 and AMP-activated protein kinase. Metformin, a hydrophilic organic cation, is a substrate of two main organic cation transporters (OCT): OCT1 and OCT2, both responsible for hepatic uptake and renal excretion of metformin. The preferential hepatic action of metformin is explained by the predominant expression of organic cation transporters 1 (OCT1) (7). The SLC22A1 gene encoding for OCT1, highly polymorphic could affect the hepatic uptake of metformin and thus explain interindividual variations in the metformin efficacy and tolerance (7, 8). The presence of at least one of the four reduced-function SLC22A1variants (R61C, G401S, M420 and/or G465R) mitigates metformin’s glycemic effects probably because of alteration in intracellular metformin transport (9). Previous studies showed that reduced-function SLC22A1 polymorphisms in humans impair the glucose-lowering effect of metformin during an oral glucose tolerance test (10, 11). A study conducted on healthy individuals showed that R61C, G401S, 420del and G465R polymorphisms of the SLC22A1 gene reduced hepatic uptake of metformin (10). Another study showed that hepatic exposure to metformin after oral administration was significantly reduced in individual with M420del and R61C variants of SLC22A1 (11). Over the past decade, the pharmacogenetic of metformin has been investigated in several studies around the world, in different ethnic populations. The majority of the studies were carried out in Asia and Europe (12–15). Many of these studies have identified polymorphisms of the SLC22A1 gene, but the reports on the effects of OCT1 polymorphisms on metformin-related therapeutic response are still contradictory (7). Some polymorphisms were associated with a lower response to metformin and others with a higher frequency of gastrointestinal adverse events (GAE) such as abdominal discomfort and diarrhea (4, 8, 16, 17). A study on the secondary effects of metformin on 251 metformin-intolerant and 1951 tolerant individuals by Genetics of Diabetes and Audit Research Tayside Study (GoDARTS) researchers demonstrated that the presence of two additional reduced-function alleles of G401S increases the likelihood of metformin intolerance, thus inducing the accumulation of metformin in enterocytes (18). A meta-analysis aimed to evaluate the associations between OCT genetic polymorphisms and metformin response and intolerance in individuals with T2D, showed that the SLC22A1 rs622342 and the GG genotype of the SLC22A1 rs628031polymorphism were associated with a reduction in HbA1c level and fasting plasma glucose level respectively (15). This meta-analysis found no statistical association between the studied genetic variants and metformin intolerance. The aims of our study were to evaluate the efficacy of metformin and the digestive tolerance to metformin in 73 newly-diagnosed type 2 diabetic patients based on M420del (rs72552763), R61C (rs12208357) and G401S (rs34130495) polymorphism of SLC22A1 gene encoding for OCT1.

## Materials and methods

The study was conducted at the Endocrinology Department of La Rabta University Hospital in Tunis. Patients older than 18 years old, with a non-treated newly diagnosed T2D were included between January 2019 and April 2020. Pregnant and lactating women, patients with heart failure, respiratory failure, chronic liver disease, severe chronic kidney disease, endocrine disorders or hormonal therapy, infection or signs of insulinopenia requiring insulin therapy were not included. All patients underwent a physical examination (weight, height, waist circumference), a dietary education and biological assessment (blood glucose, insulinemia, HbA1c, creatinine, total cholesterol, HDL cholesterol, triglycerides). After that, chlorydrate of metformin was progressively introduced: 850 mg per day on the first week then 1700 mg per day. All patients received at inclusion all the treatment for the 3 months of the study. The genetic study was carried out at the Biochemistry department of the Rabta Hospital. OCT-11258-1260delATG (M420del), OCT-1 181C>T (R61C) and OCT-1 1201G>A (G401S) genotypes were studied in all included patients. After blood centrifugation, the cell cap was kept at -20°C. Deoxyribonucleic Acid (DNA) was extracted by the salting out technique. Genotyping was performed by Polymerase Chain Reaction (PCR) followed by Restriction Fragment Length Polymorhism (RFLP). The amplified products were digested with specific restriction enzymes (HhaI, NlaIII, Sau96I), then separated by electrophoresis on agarose gel and visualized by ultraviolet irradiation. Hybridization temperature was 58 degrees Celsius. We determined the frequency of mutated allele and wild allele of the three variants in each nucleotide, genotype frequencies and the haplotypic frequencies of each genetic variant (**Table 1**).

**Table 1:** Genetic polymorphisms of the SLC22A1 gene studied.

SLC22A1 polymorphism	Primer sequence (5’→3’)	Hybridization temperature	Amplified Fragment size	Restriction enzyme	Digested fragment size Of the mutated allele
R61C (rs12208357)	F: TGATCAGATGGCCACGTGCATT	58°C	607 pb	*HhaI*	26, 100, 120, 311 pb
	R: GAGCCGGGCGTGTCATACAC				
M420del (rs72552763)	F: CTCAGGTTCACGGACTCTGTGCT	58°C	381 pb	*NlaIII*	6, 48,114, 213 pb
	R: CACTGTGCACGGCCCCTCAAT				
G401S (rs34130495)	F: CTCAGGTTCACGGACTCTGTGCT	58°C	381 pb	*Sau96I*	14, 110, 257 pb
	R: CACTGTGCACGGCCCCTCAAT				

The second visit was scheduled after 3 months. Patients underwent the same clinical and biological assessment. Adherence to metformin and common metformin-induced GAE were recorded during the anamnesis. According to the HbA1c decrease, two study groups were defined: group 1 (HbA1c decrease ≥1% after 3 months) and group 2 (HbA1c decrease <1% after 3 months). The metformin induced GAE were defined as the occurrence of any of the following symptoms during metformin therapy: bitter taste, epigastralgia, abdominal bloating, nausea, vomiting or diarrhea. Patients were divided into two groups: patients who did not develop GAE and patients who developed at least one GAE after metformin initiation. We compared the frequency of OCT1 genotypic variants and haplotypes according to metformin efficacy and digestive tolerance.

### Statistical analysis

We used Pearson’s Chi-two test and the exact bilateral Fisher test to compare qualitative variables. For the comparison of quantitative variables between 2 independent samples, we used Student’s test. Parameters with p <0.2 in univariate analysis were included as covariates into multivariate analysis using the binary logistic regression method. The genetic polymorphisms were introduced as covariate into the final model regardless of their degree of significance in univariate analysis.

## Results

Ninety-five T2D patients treatment naïve were included. Twenty-two patients were excluded: two patients developed diabetic ketosis and 20 patients were lost to follow up. Thus, the data of 73 patients were analyzed: 33 women and 40 men, mean age 53 ± 10.4 years (27-76).

SLC22A1 gene polymorphisms analysis showed that the M420del polymorphism was the most common mutated allele. The frequencies of the mutated homozygous genotypes (mm) were 15.1%, 5.5% and 4.1% for the M420del, G401S and R61C variants respectively. The haplotypes the most frequently found in our population were: (NoDel)CG (40.5%) and (Del)CG (26.1%). After 3 months of treatment with metformin, there was a significant decrease in fasting blood glucose (from 8.9 mmol/l to 6.9 mmol/l p <0.001) and HbA1c (from 8.7% to 7.1% p<0.001).

Male gender, higher HbA1c and higher fasting blood glucose levels were significantly associated with the efficacy of metformin (**Table 2**).

**Table 2:** Patients characteristics: baseline data according to metformin efficacy.

Patient’s characteristics	Metformin efficacy		P
	Yes (N=39)	No (N=34)	
Age (years)	53± 11	53 ± 9.8	NS
Women (%)	33	59	0.029
Poor therapeutic adherence (%)	14	86	0.029
Gastrointestinal adverse events (%)	37	63	0.032
Weight (Kg)	85.8 ± 13.8	83.6 ± 11.2	NS
Body mass index (kg/m^2^)	31 ± 5	31 ± 5	NS
Waist circumference (cm)	106 ± 12	105 ± 9	NS
Fasting blood glucose (mmol/l)	11.2 ± 3	8.2 ± 2.4	<10^-3^
HbA1c (%)	9.8 ± 1.7	7.4 ± 1.2	<10^-3^
Creatinine (mg/l)	8.5 ± 1.6	7.7 ± 1.6	0.038
Total cholesterol (mmol/l)	4.6 ± 1	4.6 ± 0.7	NS
HDL-cholesterol (mmol/l)	0.98 ± 0.18	1.13 ± 0.25	0.005
LDL-cholesterol (mmol/l)	3.01 ± 0.21	2.8 ± 0.85	NS
Triglycerides (mmol/l)	1.07 ± 1	1.68 ± 0.84	NS
Insulinemia (µU/ml)	7.8 ± 4.8	8.2 ± 4.2	NS
HOMA index*	3.9 ± 2.2	3 ± 2.2	0.08
M420del (%) (WW/Wm/mm)**	38.5/48.7/12.8	38.2/44.1/17.6	0.83
R61C (%) (WW/Wm/mm)**	69.2/28.2/2.6	67.6/26.5/5.9	0.77
G401S (%) (WW/Wm/mm)**	66.7/30.8/2.6	64.7/26.5/8.8	0.49

* $HOMA\;index:\;\frac{\left(fasting\;glucose\;\left[mmol/l\;]\;x\;fasting\;insulinemia[µU/ml\right]\right.\;}{22.5}$ **W, wild allele; m, mutated allele.

M420del, R61C, G401S genetic variants and haplotypes of SLC22A1were comparable between the two groups (**Tables 2**, **3**). The decrease in fasting blood glucose was significantly lower in the presence of the M420del mutant variant (1.7mmol/l versus 3.19 mmol/l; p=0.03). The logistic regression analysis including gender, poor therapeutic adherence, GAE, fasting blood glucose, HbA1c, creatinine, HDL cholesterol, HOMA index and genetics parameters showed that initial HbA1c was the only factor associated with metformin efficacy.

**Table 3:** SLC22A1 haplotypes frequencies according to metformin efficacy and gastrointestinal intolerance.

Haplotypes (%)	Metformin efficacy		p	Metformin intolerance Yes		P
	Yes (N=39)	No (N=34)		Yes (N=27)	No (N=46)	
**(NoDel)CG**	47.1	33	NS	46.7	30	NS
**(Del)CG**	23.4	29.4	NS	25.3	26.6	NS
**(NoDel)TG**	5.5	13.6	**0.08**	8.9	12.4	NS
**(Del)CA**	7.7	8.4	NS	7.7	10.9	NS
**(NoDel)CA**	5.1	10.2	NS	2.9	13.9	**0.02**
**(NoDel)TA**	5.2	3.5	NS	4.6	3	NS
**(Del)TG**	6	1.9	NS	3.9	3.2	NS

Metformin induced GAE were reported by 27 patients (37%). Diarrhea was the most common side effect. Patients with metformin induced GAE were more likely to be women and to have a history of drug digestive intolerance than those without GAE (**Table 4**).

**Table 4:** Patient’s characteristics: baseline data according to metformin induced gastrointestinal adverse events.

Patient’s characteristics	Gastrointestinal adverse events		P
	No (N=46)	Yes (N=27)	
Age (years)	53± 9	53 ± 11	NS
Women (%)	34.8	63	0.02
History of drug allergy (%)	8.7	18.5	NS
History of digestive Intolerance (%)	4.3	18.5	0.047
History of gastrointestinal symptoms (%)	6.5	14.8	NS
BMI (kg/m^2^)	31.1 ± 4.7	30.4 ± 4.7	NS
Waist circumference (cm)	106 ± 11	104 ± 10	NS
Fasting blood glucose (mmol/l)	10.1 ± 3.4	9.4 ± 3.3	NS
HbA1c (%)	8.8 ± 1.9	8.5 ± 2	NS
Cholesterol total (mmol/l)	4.6 ± 1	4.6 ± 0.7	NS
HDL-cholesterol (mmol/l)	1 ± 0.2	1.13 ± 0.2	0.057
LDL-cholesterol (mmol/l)	2.8 ± 1	2.9 ± 0.6	NS
Triglycerides (mmol/l)	1.9 ± 1.1	1.4 ± 0.5	0.025
M420del (%) (WW/Wm/mm)*	43.5/39.1/17.4	29.6/59.3/11.1	0.25
R61C (%) (WW/Wm/mm)*	67.4/30.4/2.2	70.4/22.2/7.4	0.45
G401S (%) (WW/Wm/mm)*	76.1/17.4/6.5	48.1/48.1/3.8	0.02

*W, wild allele; m, mutated allele.

There was significant association between G401S variants and metformin induced GAE. This was not the case for R61C and M420del polymorphisms (**Table 4**). The presence of at least one G401S mutated allele (Wm or mm) was associated with higher risk for developing GAE (odds ratio = 1.6, 95% confidence interval 1.03–2.66, P = 0.01). Haplotype (NoDel) CA was also significantly associated with the risk of developing GAE (**Table 3**).

In multivariate analysis including gender, history of drug digestive intolerance, HDL cholesterol, triglycerides and genetics parameters, G401S polymorphism was the only factor independently associated with metformin-induced GAE; adjusted OR= 3.4 (IC 95%:1-11.2; p=0.04).

## Discussion

Our study analyzed the relationship between genetic polymorphism of the SLC22A1gene and metformin efficacy and metformin-induced GAEs in newly diagnosed naïve T2D patients. The analysis of SLC22A1 gene polymorphisms in 73 Tunisian patients with newly T2D showed that M420del and R61C, two established loss- of-function variants in SLC22A1, and G401S variant didn’t impair metformin efficacy. Logistic regression found that higher baseline HbA1c was the only associated factor with the response to metformin. Higher Baseline A1c was reported as a factor associated with a better response to metformin and thus a more decrease in HbA1c (19, 20). In our study, we found also an association between G401S OCT1 variants, Haplotype (NoDel) CA and metformin-induced GAEs. These results confirm recent findings related to the role of OCT1 in metformin efficacy and intolerance. Most studies evaluating the effects of SLC22A1 gene polymorphisms on the therapeutic response to metformin in patients with type 2 diabetes (T2D) have focused on European, Asian, and Caucasian populations (12–14). Few studies have investigated these associations in African populations (7, 15). One study was conducted in Egypt and examined the distribution and impact of OCT1 and other gene polymorphisms on metformin efficacy in newly diagnosed T2D patients. The authors concluded that the G401S variant of OCT1 may play a potential role in influencing metformin response (21).

Genetic information plays a role in predicting clinical outcomes. The ultimate objective of pharmacogenetics is to enable personalized medicine, particularly in the management of type 2 diabetes (T2D), by accounting for interindividual genetic variability (22). Metformin is a highly hydrophilic molecule and does not undergo passive diffusion across cell membranes. Instead, its absorption and cellular uptake depend on transporters such as OCT1, which is predominantly expressed in hepatic and intestinal cell membranes (10, 23, 24). To date, more than 34 polymorphisms in the SLC22A1 gene have been studied. The frequencies of these variants differ substantially across ethnic groups (8). For instance, the rs12208357 (R61C) polymorphism has been reported to occur at frequencies ranging from 8% to 89% in different populations (10, 21). The minor allele frequency of rs72552763 (M420del) ranges from 18% to 28% in Caucasians and exceeds 90% in Japanese and Chinese populations (25). The rs34130495 (G401S) polymorphism was found to have a frequency of 4.4% in a Danish cohort (26). Seitz et al. conducted a global population analysis of OCT1 variants across 52 population groups worldwide. Their findings revealed significant interethnic variability in OCT1 activity, with a strong correlation between genetically determined loss of OCT1 function and variations in metformin pharmacokinetics and therapeutic efficacy (27). Similarly, a systematic review involving 1,079 individuals from 53 different ethnic populations demonstrated high genetic variability in OCT1. The absence of OCT1 activity was found to be very rare in East Asian populations but was notably common in certain South American populations (28). 

Several studies have reported no significant association between clinical and biological parameters such as age, gender, BMI, serum creatinine, insulinemia, and HOMA index and the therapeutic response to metformin, suggesting that genetic variation may play a more critical role in metformin pharmacokinetics (29, 30). In animal models, deletion of the SLC22A1 gene in mice led to a significant reduction in hepatic accumulation of metformin (10). Similarly, in humans, some OCT1 polymorphisms have been associated with reduced metformin effectiveness (31). Shu et al. conducted a study in a cohort of 20 healthy individuals carrying reduced-function or non-functional OCT1 variants (R61C, G401S, 420del, or G465R). In these subjects, blood glucose levels were significantly higher during the oral glucose tolerance test (OGTT). Additionally, the area under the curve for metformin concentration and the maximum plasma concentration were elevated, while the volume of distribution was reduced compared to individuals with wild-type alleles. These findings suggest impaired hepatic uptake of metformin in individuals with these SLC22A1 gene variants (10). Another study further demonstrated that hepatic exposure to metformin following oral administration was significantly reduced in individuals carrying the M420del and R61C variants, independent of circulating plasma metformin levels (32).

The M420del polymorphism is the most common SLC22A1 genetic variant, characterized by a three-base-pair deletion at position 420 (28). This polymorphism does not alter the membrane localization of OCT1 and the exact mechanism of OCT1 reduced function remains unknown. Evidence suggests that the functional impairment caused by M420del is substrate-dependent. This variant has been shown to reduce metformin absorption by up to 60% (28, 33). Three large studies have specifically investigated the impact of the M420del polymorphism on metformin response. Consistent with our findings, all three studies reported no significant association between M420del and the therapeutic response to metformin (34–36). In the Danish study, the reduction in HbA1c was comparable across different genotypes at both 6 and 24 months following metformin initiation (34). Similarly, the Scottish cohort found no correlation between M420del and metformin efficacy (35). Mahrooz et al. reached the same conclusion in a study involving 108 Iranian T2D patients (36).

The polymorphism R61C consists of a substitution of the arginine by cysteine at position 61. This variant results in the retention of OCT1 proteins within the endoplasmic reticulum and a consequent reduction in their expression on the cell membrane (28, 37). In an in vitro functional assay, Kawoosa et al. demonstrated that the R61C and G401S genotypes were associated with decreased pAMPK expression and reduced metformin transport compared to wild-type OCT1 (37). Consistent with the findings of the GoDARTS study (18), Mostafa- Hedeab G. et al. reported no significant association between R61C variant and glycemic response to metformin (21).

The G401S polymorphism, which consist of the substitution of glycine by serine at position 401, results in a loss of OCT1 transporter activity. This impairment affects the transport process without altering the membrane localization of the protein (8, 28, 37). Several studies have evaluated the association between the G401S polymorphism and metformin response (18, 32). A study involving 371 Danish individuals found a significant association between this variant and a reduction in HbA1c levels following metformin therapy (32). The same group had previously demonstrated that individuals carrying mutated alleles, grouped into haplotypes including G401S, exhibited significantly lower plasma concentrations of metformin (20).

Gastrointestinal symptoms are the most common adverse events associated with metformin, affecting approximately 30% of patients (38). The primary symptoms include diarrhea, being the most frequently reported, nausea, epigastric pain, and abdominal discomfort (39). Several mechanisms have been proposed to explain metformin-induced GAEs, including alterations in gut microbiota, increased turnover of intestinal glucose and bile acids, and elevated levels of glucagon-like peptide 1 (GLP-1) (40, 41). Additionally, vitamin B12 deficiency resulting from long-term metformin use and the presence of asymptomatic chronic gastritis prior to treatment have also been implicated (42, 43). GAEs have been associated with loss-of-function variants in the SLC22A1 gene (8, 17). A reduction in OCT1 activity in enterocyte membranes may lead to higher local concentrations of metformin within the intestinal tract, potentially disrupting the balance of incretins, ghrelin, bile acids, and serotonin, and thereby triggering gastrointestinal symptoms (7). In both Asian and Caucasian populations, the M420del variant has been linked to an increased incidence of GAEs; however, no significant association was observed in European populations (10, 44). Dujic et al. demonstrated that the presence of one or two reduced-function alleles—specifically M420del and R61C—was significantly associated with GAEs compared to individuals with wild-type alleles (4). In contrast, a study conducted by Tarasova et al. involving 246 patients with type 2 diabetes did not find a significant association between the M420del variant and metformin-induced GAEs (35). The G401S polymorphism has not been extensively studied as an isolated variant but has been examined within haplotypic associations. In our population, the G401S variant was significantly associated with metformin intolerance, with an odds ratio (OR) of 1.6 [95% CI: 1.03–2.66; p = 0.01]. Multivariate analysis confirmed G401S as an independent risk factor for digestive intolerance to metformin. Furthermore, the (NoDel) CA haplotype—comprising a single mutated G401S allele—was also associated with poor gastrointestinal tolerance. These findings support previous suggestions that the likelihood of metformin intolerance increases with the number of reduced-function OCT1 variants (18, 45). In the GoDARTS study, which included 251 patients intolerant to metformin, the presence of two reduced-function SLC22A1 alleles (R61C, M420del, or G401S) was significantly associated with gastrointestinal intolerance to metformin, with an odds ratio (OR) of 2.41 [95% CI: 1.48–3.93], compared to individuals with one or no deficient alleles (18). Similarly, in the Dujic’s study, a positive correlation was observed between the number of mutated alleles and the risk of metformin-induced digestive symptoms [OR = 2.31, 95% CI: 1.07–5.01] (45).

Although the prospective design of our study allowed for comprehensive collection of clinical and biological data, the sample size was relatively small. This limitation was primarily due to the COVID-19 pandemic, which restricted patient access to follow-up visits and contributed to a high rate of loss to follow-up. Additionally, we opted to stop patient enrollment before the onset of Ramadan in May 2020 to avoid the confounding effects of fasting on metabolic parameters. Two further limitations of our study include the absence of genetic analysis for other OCT1 variants, such as G465R and M408V, due to the unavailability of the corresponding restriction enzymes in Tunisia and budget constraints. Moreover, the lack of metformin pharmacokinetic data prevented us from exploring the relationship between metformin plasma concentrations and OCT1 polymorphisms.

Beyond metformin, gene polymorphisms in membrane transporter proteins may also influence the metabolism and efficacy of other antidiabetic medications, such as rosiglitazone and repaglinide (45). In the future, more extensive pharmacogenomic research is needed, particularly concerning newer hypoglycemic agents, to support the integration of genetic profiling into individualized treatment strategies for type 2 diabetes mellitus (T2DM). 

## Conclusions

The M420del and R61C variants did not appear to be associated with either metformin efficacy or GAEs in our study. In contrast, the G401S polymorphism may be implicated in the development of metformin-induced GAEs. Given their role in hepatic uptake of metformin, polymorphisms in the SLC22A1 gene have been reported in some studies as contributing to interindividual variability in both the efficacy and tolerance of metformin. However, the role ofOCT1in the pharmacogenetics of metformin response and intolerance remains controversial. These inconsistencies across studies may stem from differences in the frequency of genetic variants, as well as genetic and environmental heterogeneity among study populations—highlighting the need for caution when extrapolating findings across ethnically diverse groups. While our study and others did not observe a significant effect of SLC22A1 polymorphisms on metformin efficacy, this does not definitively exclude their potential influence. A more comprehensive analysis of SLC22A1 variants is necessary to fully understand their impact on metformin pharmacodynamics. Future large-scale prospective studies incorporating a broader range of polymorphisms and detailed pharmacokinetic assessments would be valuable in optimizing the personalized use of metformin in patients with type 2 diabetes.

## Data Availability

The original contributions presented in the study are included in the article/supplementary material, further inquiries can be directed to the corresponding author/s.
